# Targeted next-generation sequencing of cancer genes in poorly differentiated thyroid cancer

**DOI:** 10.1530/EC-17-0290

**Published:** 2017-11-13

**Authors:** Tiemo S Gerber, Arno Schad, Nils Hartmann, Erik Springer, Ulrich Zechner, Thomas J Musholt

**Affiliations:** 1 Endocrine Surgery SectionDepartment of General, Visceral and Transplantation Surgery, University Medicine, Mainz, Germany; 2 Department of PathologyUniversity Medicine, Mainz, Germany; 3 Institute of Human GeneticsUniversity Medicine, Mainz, Germany

**Keywords:** thyroid, endocrine cancers, rare diseases/syndromes

## Abstract

Poorly differentiated thyroid carcinoma (PDTC) is a rare malignancy with higher mortality than well-differentiated thyroid carcinoma. The histological diagnosis can be difficult as well as the therapy. Improved diagnosis and new targeted therapies require knowledge of DNA sequence changes in cancer-relevant genes. The TruSeq Amplicon Cancer Panel was used to screen cancer genomes from 25 PDTC patients for somatic single-nucleotide variants in 48 genes known to represent mutational hotspots. A total of 4490 variants were found in 23 tissue samples of PDTC. Ninety-eight percent (4392) of these variants did not meet the inclusion criteria, while 98 potentially pathogenic or pathogenic variants remained after filtering. These variants were distributed over 33 genes and were all present in a heterozygous state. Five tissue samples harboured not a single variant. Predominantly, variants in *P53* (43% of tissue samples) were identified, while less frequently, variants in *APC*, *ERBB4*, *FLT3*, *KIT*, *SMAD4* and *BRAF* (each in 17% of tissue samples) as well as *ATM*, *EGFR* and *FBXW7* (each in 13% of tissue samples) were observed. This study identified new potential genetic targets for further research in PDTC. Of particular interest are four observed *ERBB4* (alias *HER4*) variants, which have not been connected to this type of thyroid carcinoma so far. In addition, *APC* and *SMAD4* mutations have not been reported in this subtype of cancer either. In contrast to other reports, we did not find *CTNNB1* variants.

## Introduction

Poorly differentiated thyroid carcinoma (PDTC) represents an aggressive variant of thyroid cancer that predominantly arises from the differentiated variants of papillary and follicular thyroid carcinoma (PTC and FTC, respectively) but occasionally from normal follicular cells (1, 2). The incidence varies between 2–3% and 15%, depending on geographical location (3). The Turin consensus, which is based on histological growth pattern, nuclear features, mitosis, necrosis and convoluted nuclei, offered an algorithm to diagnose this entity (4). However, even with this consensus paper published in 2007, PDTC still represents a challenging diagnosis for pathologists and clinicians. Molecular changes in PDTC are heterogeneous. No exclusive mutation has been identified that could potentially facilitate the differentiation process (5, 6, 7). The advanced cancer stage and the impaired or complete lack of radioactive iodine uptake drive the search for effective therapeutic alternatives such as targeted therapies. Although Landa and coworkers (1) as well as Xu and Ghossein (8) reported an extensive investigation on PDTC, we still need to learn more about the driving molecular alterations.

Using a next-generation sequencing (NGS) approach, we screened 23 PDTC for variants in 48 cancer-relevant genes. The main goal addressed in this exploratory study was to characterise novel genetic changes as potential targets for further research as well as establishing the NGS technique in our laboratory.

## Materials and methods

### Patients and tissue acquisition

Patients treated in the Department of General, Visceral und Transplantation Surgery at University Medical Centre, Mainz, Germany between 2007 and 2015 were included in the study. The study was approved by Ethics Committee of the medical association of Rheinland Pfalz. Consent has been obtained from each patient or subject after full explanation of the purpose and nature of all procedures used. Archival tissue from the patients was retrieved and reviewed using the Turin consensus algorithm, in the course of which necrosis, the number of mitotic cells per ten high-powered fields (mitotic index) and the extrathyroidal and vascular invasion were determined. The samples were also immunohistochemically stained for thyroglobulin, thyroid transcription factor-1 and Ki-67. We did not analyse matched pairs of individual tumour-normal tissue, but only PDTC itself. Each histological slide was diagnosed by an experienced pathologist. Questionable samples were not included in this study. Twenty-five of 32 reviewed patients fulfilled all criteria to diagnose a PDTC. Furthermore, we assessed if the samples also fulfilled the Memorial Sloan-Kettering Cancer Center (MSKCC) criteria for PDTC as proposed by Hiltzik and coworkers (9). These were defined by the presence of ≥5 mitotic cells per ten high-powered fields and/or fresh tumour necrosis. The fraction of malignant cells in tumour tissue was determined by visual estimation of the percentage of malignant cells (tumour cell to non-tumour cell). An overview of the patient data is presented in Table 1. Routine Sanger sequencing was performed on the *BRAF V600E* and *BRAF* wild-type V600 gene locus.

**Table 1:** Individual characteristics of cases.

**No.**	**Clinical and patient data**								**Pathology**				
					Metastasis				Vessel invasion				
	Sex	Age	Tumour size (cm)	T category	Node	Distant	Treatment	Local complications^a^	Lymph	Blood	ETE	TCF	P53 status
1	m	76	8	III	No	Mx	TT + RAI	None	Yes	Yes	Yes	60%	Variant
2	w	78	8.5	III	Yes	Lung, bone	TT + RAI	Dyspnoea, dysphagia	Yes	Yes	No	90%	Wt
3	w	65	7.7	IVa	Yes	Lung, bone	TT + RAI + PT	Dyspnoea, dysphagia	Yes	Yes	Yes	90%	Variant
4	w	43	2.8	II	No	Mx	TT	None	No	No	Yes	90%	Wt
5	m	64	6.3	III	Yes	Mx	TT	None	Yes	No	Yes	70%	Wt
6	w	81	NA	NA	NA	NA	NA	NA	No	Yes	NA	50%	Variant
7	m	73	9	IVa	Yes	M0	TT + CTx + PT	None	Yes	Yes	Yes	60%	Wt
8	w	77	7.5	IVa	No	M0	TT + RAI	Dyspnoea	Yes	Yes	Yes	80%	Wt
9	m	64	9.4	III	No	Bone	TT + CTx	Infiltration trachea	No	Yes	Yes	80%	Variant
10	m	65	8.5	III	No	M0	TT + RAI	Dyspnoea	No	Yes	Yes	90%	Wt
11	w	27	2.2	II	No	M0	TT	None	No	No	No	50%	Variant
12	m	72	NA	IV	Yes	Mx	TT + CTx + PT + RAI	Infiltration oesophagus	Yes	Yes	Yes	90%	Wt
13	m	86	6.7	III	Yes	Lung	TT + CTx	None	Yes	No	Yes	70%	Wt
14	m	70	4	IVa	No	Lung	TT + PT + RAI	Infiltration trachea	No	Yes	Yes	70%	Variant
15	w	53	1.2	II	No	M0	TT	None	No	No	No	90%	Wt
16	w	59	1.5	III	Yes	Lung, bone	TT + PT + RAI	Infiltration oesophagus and trachea	Yes	Yes	Yes	60%	Variant
17	m	67	NA	IVa	Yes	Lung, bone	TT + PT + CTx + RAI	Infiltration oesophagus and trachea	Yes	No	NA	90%	Variant
18	w	79	NA	III	Yes	M0	TT + RAI	None	No	Yes	NA	80%	Variant
19	m	66	2.5	III	Yes	Lung, liver	TT + CTx + PT + RAI	Infiltration larynx	Yes	No	Yes	50%	Wt
20	w	57	0.9	IVa	Yes	Lung	TT + PT	Infiltration oesophagus and trachea	No	Yes	Yes	60%	Wt
21	w	67	4	III	No	M0	TT + RAI	None	No	Yes	Yes	90%	Wt
22	m	68	4.8	III	Yes	Lung, liver	TT + CTx	Dyspnoea	Yes	Yes	Yes	60%	Variant
23	m	76	11	IVb	Yes	Mx	TT + PT + RAI	Infiltration trachea	Yes	Yes	Yes	70%	Wt

^a^During any time after surgical treatment.

CTx, Chemotherapy; ETE, extrathyroidal extension of tumour; NA, not available; PT, percutaneous radiotherapy; RAI, radioactive iodine treatment; TCF, tumour cell fraction (cell-cell ratio); TT, total thyroidectomy (usually with central node dissection); Wt, wild type.

### DNA extraction and sample quality control

Genomic DNA was extracted from the formalin-fixed and paraffin-embedded tumour material. To reduce contamination, microscope slides of the tumour stained with haematoxylin–eosin were used to select areas for macrodissection. The selected material was scratched off from 2 μm thick unstained slides. It was deparaffinised with xylene and ethanol and digested by proteinase K in lysis buffer overnight at 56°C. The samples were purified with MPC Protein Precipitation Solution (Biozym Scientific GmbH, Oldendorf, Germany) and precipitated with isopropanol. The DNA was quantified using a spectrophotometer to measure the absorbance at 260 nm. The DNA quality was tested with the FFPE QC Kit for the TruSeqAmplicon Cancer Panel (Illumina, San Diego, CA, USA) using the manufacturer’s instructions. Table 2 for a full list of covered genes. Two samples failed the quality control criteria, resulting in 23 analysed PDTCs.

**Table 2:** Truseq amplicon cancer panel gene list.

**Illumina TSCAP**			
ABL1	ERBB4	JAK2	PIK3CA
AKT1	FBXW7	JAK3	PTEN
ALK	FGFR1	KDR	PTPN11
APC	FGFR2	KIT	RB1
ATM	FGFR3	KRAS	RET
BRAF	FLT3	MET	SMAD4
CDH1	GNA11	MLH1	SMARCB1
CDKN2A	GNAQ	MPL	SMO
CSF1R	GNAS	NOTCH1	SRC
CTNNB1	HNF1A	NPM1	STK11
EGFR	HRAS	NRAS	TP53
ERBB2	IDH1	PDGFRA	VHL

### Library preparation and MiSeq sequencing

Sequencing libraries were prepared using the TruSeq Amplicon Cancer Panel (Illumina) with the MiSeq Reagent Kit, v2 according to the manufacturer’s instructions. The MiSeq System (Illumina) was used to launch the massively parallel sequencing process to capture the exons of 48 genes with 212 amplicons.

### Data analysis

Using the TruSeq Amplicon Workflow on the MiSeq Reporter, the data were demultiplexed and aligned to the human reference genome hg19. The output was generated in the variant caller format. BaseSpace Variant Studio, v2.2.4 (Illumina) was used for post processing. This programme could integrate information about called variants from the Cosmic (v65) and ClinVar (v05.09.2013) scientific databases. The application uses the Variant Effect Predictor (VEP) tool for Annotation and Analysis of coding sequence changes. VEP also harnesses algorithms such as Polymorphism Phenotyping 2 (PolyPhen-2) and Sorting Intolerant From Tolerant (SIFT).

To eliminate false positive called variants we used a specific algorithm. First, all variants needed at least a coverage above 20, a sequencing depth of at least 100 and a mutation frequency of 10%, resulting in a threshold of 10 counts for a variant. Second, all variants needed to be nonsynonymous and to have a genotype quality of at least 30. Third, all variants needed to be marked as potentially deleterious in SIFT, as potentially damaging in PolyPhen, as potentially pathogenic or pathogenic in ClinVar or listed in Cosmic. Genes were named in accordance with the guidelines for human gene nomenclature (Supplementary Table 1, see section on supplementary data given at the end of this article; (10)).

## Results

**Figure 1:**
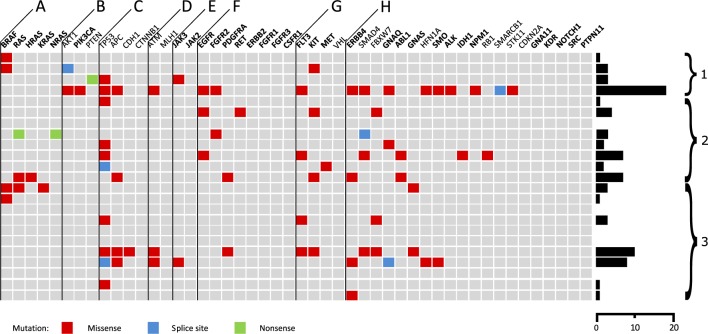
The columns show the analysed genes sorted by the molecular pathway affected. Oncogenes are displayed bold while tumor suppressor genes are displayed normal. (A) RAS, (B) PIK3, (C) Wnt, (D) DNA damage control, (E) STAT, (F) RAS + PIK3, (G) RAS + PIK3 + STAT, (H) others. The rows show the 23 PDTC specimens. The variant effect is shown by the colour of the mutation. On the right side of the figure is indicated the mutational burden. (1) PDTC with coexisting PTC. (2) PDTC with coexisting follicular tumour components. (3) PDTC only.

Eighteen tissue samples of PDTCs showed a total number of 98 variants as presented in Fig. 1. All variants were present in a heterozygous state. Ninety-eight genetic changes were single-nucleotide variants (90 missense variants, two nonsense variants, and 6 splice site variants), while one was a deletion leading to a frameshift. The sequencing depth ranged between 101 and 10258 (median 161). The mutation frequency had a median of 13.2. The coverage varied between 21 and 280 with a median of 102. Eighty-seven variants were identified with a genotype quality of 100. For detailed information on genetic alterations see Supplementary Table 1.

The most commonly altered gene was *TP53*, affected in 10 of 23 samples (43%). All *TP53* variants occurred at different base positions. The *FLT3*, *SMAD4* and *BRAF* variants were detected in each of four samples. Four variants were observed in the each of the *APC*, *BRAF*, *ERBB4*, *FLT3*, *KIT* and *SMAD4* gene loci. In three of the four samples with *BRAF* variants, we found the p.V600E variant and in one, the p.G469V variant. Changes in the *ATM*, *EGFR* and *FBXW7* genes were identified in each of the three samples (13%).

One-hundred percent concordance in *BRAF* variant status was observed between NGS and routine conventional Sanger sequencing. Each tumour sample of PDTC diagnosed with the Turin consensus criteria was also classified as PDTC by the MSKCC-criteria that indicated more aggressive PDTC.

## Discussion

Two main questions drive the search for new molecular alterations of PDTC. First, what targeted therapies could add a promising approach to the treatment? Second, which mutational markers could improve the accuracy of diagnosis for advanced thyroid carcinomas? This study aims to contribute to this growing area of research by exploring 48 genes of interest. It is beyond the scope of this explorative study to prove causality or to address the progression from well-differentiated thyroid carcinoma (WDTC) to PDTC.

### *BRAF*, *TP53*, *RAS*

While tumour-initiating somatic mutations leading to papillary thyroid carcinoma have been researched to a great extent, it remains uncertain whether and to what extent particular genetic alterations participate in the tumour progression to PDTC. The Cancer Genome Atlas Research Network study has identified many oncogenic mutations for PTC. It is generally agreed today that PDTC can originate from WDTC (11).

Landa and coworkers has shown that the changes to the *BRAF* to *RAS* relationship are still preserved in PDTC (1). For these reasons, one could assume that certain driver mutations are conserved during the dedifferentiation process. In our study, we found three *RAS* variants (one each of *N-*, *K-* and *HRAS*) and three *BRAF*p.V600E variants. However, it is not the same as a *BRAF/RAS*-score based on mRNA data (11). In accordance with the aforementioned studies, there have been no *RAS* and *BRAF*p.V600E variants in the same tissue sample.

Mutations in *BRAF*, *EIF1AX*, *TP53* and *TERT* are associated with aggressive behaviour of thyroid malignancies (2, 8, 12). *BRAF* and *TP53* are associated with the dedifferentiation progress to anaplastic thyroid carcinomas (ATCs) (13). In our study, there were 10/23 cases of PDTC harbouring a *p53* variant. There was no significant difference regarding the clinical course, extrathyroidal extension or vascular invasion between *TP53*-positive tumours and the other tumours. One possible reason may be the preselection of an already aggressive subtype of cancer. Some previously published works have assessed *TP53* mutations in PDTC. They observed lower rates (8, 27 and 38%) of this variant than in our series (1, 14, 15). The variety might be related to the generally low number of patients in our study (23 tissue samples) as well as in compared series (84, 22 and 46 patients). The *TERT* and *EIF1AX* gene locations were not included in the applied cancer panel.

In contrast to previous studies, we found less RAS variants (13%) than described in the literature (20–55%) (16, 17, 18, 19, 20). These numbers are slightly lower than the value we expected. It seems plausible that a number of limitations could have influenced the obtained results. On the one hand, we used tissue that was formalin fixed and paraffin embedded for several years, which leads to the necessity of a very conservative filtering process to avoid false-positive findings from artefacts. This implies that there could be true-positive variants that have been lost in the background of artefacts. On the other hand, it is obvious that the overall small number of PDTC cases impairs conclusions based on the comparison of percentages.

### *APC, ATM, CTNNB1, EGFR* and *ERBB4*

PDTC are a genetically heterogeneous entity. In this study, we identified new potential genetic targets for further investigation. Two of these are *EGFR* (*ERBB1*) and *ERBB4*, which have not been previously reported to play a role in thyroid carcinogenesis. *ERBB1* is known to be mutated in cancers like gliomas or small-cell lung cancer and associated with the epithelial–mesenchymal transition as well as tumour invasion (21). In colon cancer, the mutation of *ERBB4* resulted in a loss of differentiation and an activation of the phosphoinositide 3-kinase (PI3K) signalling pathway (22).

Another valuable target is *APC*. Recent studies on PTC identified *APC* as well as *ATM* as potential driver mutations (11). PDTC may harbour mutations that are present in PTC as they may derive from WDTC (23). Furthermore, *APC* mutations have been observed in ATC (24). Considering these arguments, it seems possible that this mutation plays a role in PDTC carcinogenesis as well.

Somatic mutations of *CTNNB1* were discussed being associated PDTC and ATC (25). However, we detected no variants of this gene in the PDTC examined. The absence of CTNNB1 mutations supports the hypothesis of Rocha and coworkers (26) that loss of E-cadherin expression rather than β-catenin (CTNNB1) gene mutations induces the process of tumour dedifferentiation.

### *SMAD4* and *KIT*

*SMAD4* encodes a mediator protein in the transforming growth factor β (TGF-β) signalling pathway. This can lead to a loss of TGF-β-mediated growth inhibition (27). In our study, five variants affected four patients (17%). The observed mutations were scattered along the sequence of the *SMAD4* gene locus, suggesting that there is no mutational hotspot present. This locus is known to be altered in WDTC, oesophageal adenocarcinoma, pancreatic cancer and colorectal cancer (27, 28, 29). A *SMAD4* immunohistochemistry expression study has shown a correlation with poor survival in colon cancer (30). An in-depth examination of *SMAD4* status demonstrated that abnormalities in this locus occur in benign as well as in malignant thyroid tumours (15/56, 27%), which was interpreted by the authors as indicative of an early event in thyroid tumorigenesis (28). All this leads to the assumption that the presence of *SMAD4* mutations cannot differentiate between well-differentiated thyroid cancer and PDTC.

*KIT* encodes for a tyrosine kinase receptor and mediates growth regulation. Mutations in this gene are associated with haematologic diseases and gastrointestinal stromal tumours (31). *KIT* expression is reduced in PTC compared to benign nodules (32). Its assessment can improve the diagnosis in fine-needle aspiration (33, 34). There are, however, little data available for *KIT* mutational status in PDTC, although it is known that alterations in KIT transcripts increase with tumour progression from WDTC to PDTC (35, 36). In a study with 118 ATCs, none showed a *KIT* mutation (37). In our study, we detected four *KIT* variants. This discrepancy may indicate a slowing effect on cancer growth that is lost during tumour dedifferentiation to anaplastic thyroid cancer, which may be lost in favour of a more deleterious mutation in the downstream signalling pathways.

### Coexistent follicular or papillary tumours

**Figure 2:**
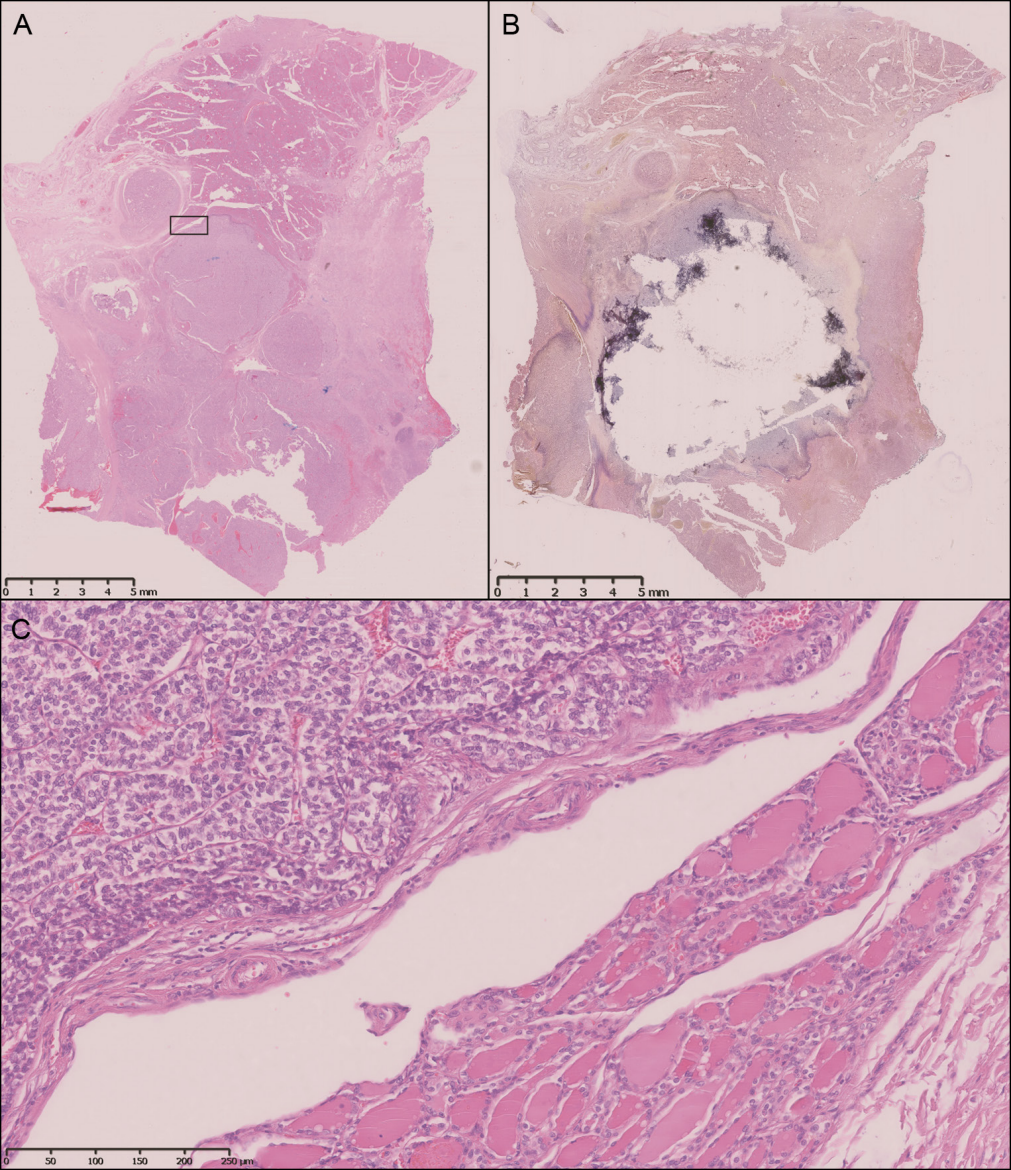
Macrodissection of a tumour with two different entities. Complete slide of the specimen before (A) and after (B) dissection of the PDTC. (C) A solid growing PDTC is demonstrated on the upper left next to a coexisting follicular thyroid tumour. Magnified clipping of a as indicated. (*A, B and C: hematoxylin-eosin stain; A and B: 5× magnification; C: 200× magnification*).

Surprisingly, we found many tumours with coexistent follicular or papillary carcinoma components (Figs 1 and 2). Eight PDTCs showed follicular coexistent components while five showed proportions with papillary carcinomas. Given the higher incidence of PTC than FTC, this could indicate a more frequent progression of FTC to PDTC. This is in accordance with the findings of Soares and coworkers (38) who emphasised that PDTC are more closely related to FTC than to PTC. The authors established their assumption on the absence of *BRAF* variants in 19 PDTC. In contrast to their results, we found three *BRAF V600E* variants. Two of the three p.V600E *BRAF* variants were in PDTC with PTC proportions. The discrepancy is most likely related to the fact that Soares and coworkers excluded PDTC with PTC components in their series.

### Methodological considerations

Five PDTCs did not show any genetic alteration at all, which implies that yet unknown driving genetic alterations exist or that the applied methods failed to detect them. According to Bozic and coworkers (39), the actual selective growth advantage of a cancer by typical mutations is much smaller than commonly assumed. The majority of genes leading to a carcinoma (or supporting its dedifferentiation consecutively) have a low mutation frequency (40). Thus, it is possible that either these PDTCs had genetic variants not covered by the NGS panel or they had a mutation frequency less than 10% and were not distinguishable from artefacts.

The results of the present study demonstrate that there are many noteworthy genes that could be responsible for the dedifferentiation of thyroid carcinoma, but a number of restrictions of our study should be mentioned. One should note that we did not analyse blood or normal tissue to discriminate between true mutations or SNP. In a detailed examination of germline mutations in tumour tissue 3% of all samples showed germline mutations (41). We can assume similar results. Given the exploratory nature of this study, the variants presented here can only serve as an indicator for more targeted studies that should consider pathogenic germline alterations.

Furthermore, it is important to emphasise that the data have not been completely validated by Sanger sequencing. Acknowledging the limitations of validation of NGS-derived variants using Sanger sequencing, we can surmise that the validation rate is precise enough for an exploratory study (42). Admittedly, contrary to the aforementioned study, we used paraffin-embedded formalin-fixed tissue. This pre-treated tissue could potentially be the source of sequencing artefacts. Following recommendations to reduce this effect, we macrodissected tumour-rich tissue, measured the quantity of DNA and excluded insufficient tissue samples (43). Given the nature of the TruSeq Amplicon Cancer Panel, we only sequenced exonic DNA with a limitation on certain genes. Due to this limitation, it was not possible to detect rearrangements of oncogenes like RET and NTRK1 or mutations of *TERT*. The latter are suspected to be connected to the disease progression of WDTC and to distinguish PDTCs from ATCs (1). However, rearrangements are rarely described in PDTC possibly because WDTC expressing RET/PTC hybrid oncogenes will usually not dedifferentiate further (44).

Even with the recent progress in the knowledge of molecular changes in PDTC, we think that our study reveals some novel variants, possibly influencing future research.

## Supplementary Material

Supporting Table 1

## Declaration of interest

The authors declare that there is no conflict of interest that could be perceived as prejudicing the impartiality of the research reported.

## Funding

This research did not receive any specific grant from any funding agency in the public, commercial or not-for-profit sector.
