# Supplementary material for: Targeted next-generation sequencing of cancer genes in poorly differentiated thyroid cancer
Source: Endocr Connect. 2017 Nov 13;7(1):47–55. doi: 10.1530/EC-17-0290 (PMC5744626; doi:10.1530/EC-17-0290)
Supplement: Supporting Table 1 [file ec-7-47-t001.pdf]

**Supplementary table 1**

| Sample Number | Tumour cell fraction | Gene | Alt Variant Freq (%) | Read Depth | Alt Read Depth | Consequence | HGVSc[a] | COSMIC Primary Site |
|---|---|---|---|---|---|---|---|---|
| **RAS-only Pathway** | | | | | | | | |
| 7 | 60% | BRAF | 20, 45 | 2959 | 605 | missense variant | NM 004333.4:c.1406G>T | oesophagus, biliary tract, skin, large intestine, lung, upper aerodigestive tract, skin, large intestine, haematopoietic and lymphoid tissue, small intestine, cervix, large intestine |
| 12 | 90% | BRAF | 28, 06 | 2648 | 743 | missense variant | NM 004333.4:c.1799T>A | skin, prostate, liver, oesophagus, adrenal gland, biliary tract, bone, soft tissue, breast, genital tract, large intestine, central nervous system, ovary, stomach, haematopoietic and lymphoid tissue, urinary tract, pancreas, autonomic ganglia, pituitary, lung, thyroid, upper aerodigestive tract, testis, eye, small intestine, endometrium |
| 13 | 70% | BRAF | 41, 08 | 1407 | 578 | missense variant | NM 004333.4:c.1799T>A | |
| 20 | 60% | BRAF | 32, 78 | 659 | 216 | missense variant | NM 004333.4:c.1799T>A | |
| 15 | 90% | HRAS | 23, 19 | 207 | 48 | missense variant | NM 005343.2:c.181C>A | skin, autonomic ganglia, lung, thyroid, upper aerodigestive tract, skin, kidney, soft tissue, urinary tract, testis, cervix |
| 7 | 60% | KRAS | 41, 21 | 1861 | 767 | missense variant | NM 033360.2:c.35G>A | oesophagus, kidney, endometrium, breast, large intestine, haematopoietic and lymphoid tissue, pancreas, lung, thyroid, liver, cervix, biliary tract, soft tissue, skin, peritoneum, central nervous system, pleura, ovary, stomach, urinary tract, autonomic ganglia, gastrointestinal tract (site indeterminate), prostate, upper aerodigestive tract, testis, eye, thymus, small intestine, salivary gland, bone |
| 21 | 90% | NRAS | 10, 32 | 155 | 16 | stop gained | NM 002524.4:c.120C>A | lung |
| **PIK3-only Pathway** | | | | | | | | |
| 11 | 50% | PIK3CA | 14, 48 | 145 | 21 | missense variant | NM 006218.2:c.2132A>C | |
| 15 | 90% | PIK3CA | 10, 74 | 149 | 16 | missense variant | NM 006218.2:c.3079G>A | |
| 1 | 60% | PTEN | 36, 44 | 450 | 164 | stop gained | NM 000314.4:c.49C>T | breast, haematopoietic and lymphoid tissue, lung, endometrium |

| | | | | | | | | |
|---|---|---|---|---|---|---|---|---|
| 11 | 50% | AKT1 | 11, 76 | 102 | 12 | missense variant | NM 001014431.1:c.143G>A | large intestine |
| 20 | 60% | AKT1 | 27, 78 | 198 | 55 | missense variant, splice region variant | NM 001014431.1:c.49G>A | endometrium, breast, large intestine, central nervous system, ovary, haematopoietic and lymphoid tissue, urinary tract, prostate, lung, thyroid |
| **Wnt Pathway** | | | | | | | | |
| 11 | 50% | APC | 19, 2 | 125 | 24 | missense variant | NM 000038.5:c.2693A>T | |
| 11 | 50% | APC | 10, 11 | 465 | 47 | missense variant | NM 000038.5:c.4033G>A | large intestine |
| 11 | 50% | APC | 10, 58 | 208 | 22 | missense variant | NM 000038.5:c.4580C>T | |
| 15 | 90% | APC | 10, 4 | 173 | 18 | missense variant | NM 000038.5:c.3893C>T | |
| 15 | 90% | APC | 10, 4 | 327 | 34 | missense variant | NM 000038.5:c.4357C>T | large intestine, upper aerodigestive tract |
| 16 | 60% | APC | 17, 65 | 170 | 30 | missense variant | NM 000038.5:c.2684C>T | large intestine |
| 16 | 60% | APC | 14, 55 | 110 | 16 | missense variant | NM 000038.5:c.4162A>T | |
| 18 | 80% | APC | 12, 61 | 111 | 14 | missense variant | NM 000038.5:c.4639G>A | biliary tract, large intestine |
| 16 | 60% | CDH1 | 27, 07 | 133 | 36 | missense variant | NM 004360.3:c.297G>C | |
| 1 | 60% | TP53 | 27, 72 | 1086 | 301 | missense variant | NM 000546.5:c.839G>C | biliary tract, skin, vagina, breast, large intestine, central nervous system, haematopoietic and lymphoid tissue, stomach, urinary tract, prostate, lung, upper aerodigestive tract, small intestine, oesophagus, endometrium, ovary, oesophagus |
| 3 | 90% | TP53 | 11, 21 | 107 | 12 | missense variant | NM 000546.5:c.909C>G | large intestine |
| 3 | 90% | TP53 | 80, 53 | 113 | 91 | missense variant | NM 000546.5:c.745A>T | breast, liver, oesophagus, large intestine, central nervous system, ovary, stomach, haematopoietic and lymphoid tissue, soft tissue, urinary tract, lung, upper aerodigestive tract, endometrium, skin, small intestine |
| 3 | 90% | TP53 | 12, 39 | 113 | 14 | frameshift variant, feature truncation | NM 000546.5:c.742delC | bone, lung |
| 3 | 90% | TP53 | 11, 21 | 107 | 12 | missense variant | NM 000546.5:c.509C>T | skin, large intestine, central nervous system, stomach, haematopoietic and lymphoid tissue, upper aerodigestive tract |
| 6 | 50% | TP53 | 10, 05 | 199 | 20 | missense variant | NM 000546.5:c.839G>A | biliary tract, skin, vagina, breast, large intestine, central nervous system, haematopoietic and |

| | | | | | | | | |
|---|---|---|---|---|---|---|---|---|
| | | | | | | | | lymphoid tissue, stomach, urinary tract, prostate, lung, upper aerodigestive tract, small intestine, oesophagus, endometrium, ovary, prostate |
| 6 | 50% | TP53 | 10, 05 | 199 | 20 | missense variant | NM 000546.5:c.818G>A | liver, cervix, oesophagus, biliary tract, fallopian tube, vulva, bone, skin, breast, penis, genital tract, large intestine, central nervous system, ovary, stomach, haematopoietic and lymphoid tissue, lung, thyroid, upper aerodigestive tract, thymus, endometrium, urinary tract, soft tissue, pancreas |
| 9 | 80% | TP53 | 72, 07 | 2338 | 1685 | missense variant | NM 000546.5:c.659A>G | liver, oesophagus, biliary tract, bone, soft tissue, endometrium, breast, peritoneum, large intestine, central nervous system, ovary, stomach, haematopoietic and lymphoid tissue, urinary tract, pancreas, prostate, lung, upper aerodigestive tract, small intestine |
| 11 | 50% | TP53 | 16 | 150 | 24 | missense variant | NM 000546.5:c.742C>T | liver, oesophagus, cervix, biliary tract, vulva, bone, soft tissue, kidney, skin, endometrium, breast, penis, large intestine, central nervous system, pancreas, ovary, stomach, haematopoietic and lymphoid tissue, urinary tract, prostate, lung, thyroid, upper aerodigestive tract, thyroid |
| 11 | 50% | TP53 | 13, 33 | 150 | 20 | missense variant | NM 000546.5:c.730G>A | liver, oesophagus, biliary tract, soft tissue, skin, endometrium, breast, large intestine, central nervous system, ovary, stomach, urinary tract, pancreas, lung, upper aerodigestive tract, adrenal gland, kidney, large intestine, prostate, haematopoietic and lymphoid tissue |
| 14 | 70% | TP53 | 37, 68 | 10258 | 3865 | missense variant, splice region variant | NM 000546.5:c.376T>A | breast, oesophagus, haematopoietic and lymphoid tissue, pancreas, prostate, central nervous system, lung, large intestine |
| 16 | 60% | TP53 | 11, 79 | 195 | 23 | missense variant | NM 000546.5:c.532C>T | skin, upper aerodigestive tract, vulva, skin, large intestine, ovary, stomach, haematopoietic and lymphoid tissue, bone, endometrium, breast, lung, thyroid |
| 17 | 90% | TP53 | 45, 01 | 1062 | 478 | missense variant | NM 000546.5:c.737T>C | biliary tract, skin, breast, large intestine, |

| | | | | | | | | |
|---|---|---|---|---|---|---|---|---|
| | | | | | | | | haematopoietic and lymphoid tissue, oesophagus, autonomic ganglia, upper aerodigestive tract, breast, lung |
| 18 | 80% | TP53 | 15, 83 | 139 | 22 | missense variant | NM 000546.5:c.1022T>A | large intestine, prostate |
| 18 | 80% | TP53 | 60, 81 | 148 | 90 | splice acceptor variant | NM 000546.5:c.376-1G>A | Skin, ovary, liver, bone, breast, large intestine, central nervous system, haematopoietic and lymphoid tissue, pancreas, prostate, upper aerodigestive tract, oesophagus, autonomic ganglia, lung, urinary tract |
| 22 | | TP53 | 28, 24 | 4210 | 1189 | missense variant | NM 000546.5:c.701A>G | breast, oesophagus, adrenal gland, cervix, large intestine, central nervous system, biliary tract, ovary, haematopoietic and lymphoid tissue, kidney, urinary tract, prostate, lung, upper aerodigestive tract, prostate, skin |
| **DNA damage control** | | | | | | | | |
| 11 | 50% | ATM | 10, 39 | 231 | 24 | missense variant | NM 000051.3:c.3964C>T | haematopoietic and lymphoid tissue |
| 11 | 50% | ATM | 18, 24 | 170 | 31 | missense variant | NM 000051.3:c.5309C>T | large intestine, haematopoietic and lymphoid tissue |
| 16 | 60% | ATM | 13, 59 | 103 | 14 | missense variant | NM 000051.3:c.5126A>T | |
| 18 | 80% | ATM | 12, 96 | 108 | 14 | missense variant | NM 000051.3:c.5038C>T | |
| **STAT Pathway** | | | | | | | | |
| 1 | 60% | JAK3 | 72, 47 | 977 | 708 | missense variant | NM 000215.3:c.2164G>A | haematopoietic and lymphoid tissue |
| 18 | 80% | JAK3 | 24, 39 | 123 | 30 | missense variant | NM 000215.3:c.2074G>A | |
| **RAS + PIK3 Pathway** | | | | | | | | |
| 10 | | RET | 12, 96 | 270 | 35 | missense variant | NM 020975.4:c.1889G>A | |
| 3 | 90% | EGFR | 29, 7 | 101 | 30 | missense variant | NM 005228.3:c.2174C>T | pleura, ovary |
| 10 | | EGFR | 12, 81 | 203 | 26 | missense variant | NM 005228.3:c.2621G>T | |
| 11 | 50% | EGFR | 11, 54 | 104 | 12 | missense variant | NM 005228.3:c.1850G>T | |
| 11 | 50% | FGFR2 | 11, 59 | 138 | 16 | missense variant | NM 022970.3:c.1627G>A | |
| 21 | | FGFR2 | 11, 18 | 170 | 19 | missense variant | NM 022970.3:c.800C>T | |
| 15 | | PDGFRA | 11, 27 | 142 | 16 | missense variant | NM 006206.4:c.1991G>T | |

| | | | | | | | | |
|---|---|---|---|---|---|---|---|---|
| 16 | 60% | PDGFRA | 20, 21 | 188 | 38 | missense variant | NM 006206.4:c.2473C>T | |
| **RAS + PIK3 + STAT Pathway** | | | | | | | | |
| 3 | 90% | FLT3 | 19, 01 | 121 | 23 | missense variant | NM 004119.2:c.1969G>A | |
| 6 | 50% | FLT3 | 11, 68 | 291 | 34 | missense variant | NM 004119.2:c.1734G>A | |
| 11 | 50% | FLT3 | 10, 53 | 133 | 14 | missense variant | NM 004119.2:c.2515G>A | |
| 11 | 50% | FLT3 | 19, 7 | 132 | 26 | missense variant | NM 004119.2:c.2503G>A | haematopoietic and lymphoid tissue |
| 11 | 50% | FLT3 | 10, 53 | 133 | 14 | missense variant | NM 004119.2:c.2455G>A | |
| 16 | 60% | FLT3 | 14, 81 | 108 | 16 | missense variant | NM 004119.2:c.2519C>A | |
| 10 | 90% | KIT | 12, 07 | 116 | 14 | missense variant | NM 000222.2:c.2582A>T | large intestine |
| 15 | 90% | KIT | 11, 69 | 154 | 18 | missense variant | NM 000222.2:c.2194G>T | |
| 16 | 60% | KIT | 10, 26 | 117 | 12 | missense variant | NM 000222.2:c.2552A>G | |
| 20 | 60% | KIT | 11, 45 | 227 | 26 | missense variant | NM 000222.2:c.2063C>T | |
| 14 | 70% | MET | 48, 9 | 7910 | 3868 | missense variant | NM 001127500.1:c.3029C>T | lung, thyroid |
| **Other molecular Pathways** | | | | | | | | |
| 3 | 90% | ABL1 | 14, 21 | 197 | 28 | missense variant | NM 007313.2:c.1018G>A | |
| 15 | 90% | ABL1 | 12, 97 | 185 | 24 | missense variant | NM 007313.2:c.1011G>A | |
| 11 | 50% | ALK | 11, 4 | 228 | 26 | missense variant | NM 004304.4:c.3758G>A | |
| 11 | 50% | ERBB4 | 15, 64 | 179 | 28 | missense variant | NM 005235.2:c.1841G>A | |
| 11 | 50% | ERBB4 | 10, 06 | 179 | 18 | missense variant | NM 005235.2:c.1766G>A | lung |
| 11 | 50% | ERBB4 | 26, 28 | 137 | 36 | missense variant | NM 005235.2:c.638G>A | |
| 11 | 50% | ERBB4 | 10, 26 | 156 | 16 | missense variant | NM 005235.2:c.316C>A | large intestine |
| 15 | 90% | ERBB4 | 10, 29 | 136 | 14 | missense variant | NM 005235.2:c.698A>G | |
| 15 | 90% | ERBB4 | 10, 22 | 137 | 14 | missense variant | NM 005235.2:c.656G>A | |
| 15 | 90% | ERBB4 | 12, 86 | 140 | 18 | missense variant | NM 005235.2:c.493G>A | |
| 18 | 80% | ERBB4 | 13, 5 | 163 | 22 | missense variant | NM 005235.2:c.497T>A | large intestine |
| 23 | 70% | ERBB4 | 52, 59 | 1603 | 843 | missense variant | NM 005235.2:c.338A>T | |
| 6 | 50% | FBXW7 | 13, 83 | 188 | 26 | missense variant | NM 033632.3:c.1363C>G | |
| 10 | 90% | FBXW7 | 11, 92 | 193 | 23 | missense variant | NM 033632.3:c.1433C>A | |
| 16 | 60% | FBXW7 | 15, 73 | 178 | 28 | missense variant | NM 033632.3:c.1709C>T | |

| | | | | | | | | |
|---|---|---|---|---|---|---|---|---|
| 16 | 60% | FBXW7 | 15, 64 | 179 | 28 | missense variant | NM 033632.3:c.1702G>A | |
| 11 | 50% | GNAQ | 14, 47 | 152 | 22 | missense variant | NM 002072.3:c.715C>T | |
| 17 | 90% | GNAQ | 28 | 150 | 42 | missense variant | NM 002072.3:c.789G>T | |
| 18 | 80% | GNAQ | 11, 83 | 169 | 20 | missense variant, splice region variant | NM 002072.3:c.738C>G | |
| 7 | 60% | GNAS | 22, 31 | 1049 | 234 | missense variant | NM 080425.2:c.2531G>A | liver, oesophagus, biliary tract, soft tissue, kidney, breast, large intestine, ovary, haematopoietic and lymphoid tissue, stomach, pancreas, pituitary, lung, thyroid, small intestine, lung |
| 16 | 60% | GNAS | 17, 65 | 102 | 18 | missense variant | NM 080425.2:c.2555A>T | |
| 11 | 50% | HNF1A | 11, 63 | 129 | 15 | missense variant | NM 000545.5:c.671C>T | |
| 18 | 80% | HNF1A | 10, 74 | 242 | 26 | missense variant | NM 000545.5:c.904C>T | |
| 3 | 90% | IDH1 | 11, 67 | 240 | 28 | missense variant | NM 005896.2:c.326G>A | |
| 11 | 50% | NPM1 | 10, 71 | 168 | 18 | missense variant | NM 002520.6:c.876G>T | |
| 3 | 90% | RB1 | 11, 01 | 109 | 12 | missense variant | NM 000321.2:c.458A>G | |
| 3 | 90% | SMAD4 | 18, 87 | 106 | 20 | missense variant | NM 005359.5:c.611C>T | lung |
| 3 | 90% | SMAD4 | 24, 39 | 123 | 30 | missense variant | NM 005359.5:c.1532C>T | |
| 11 | 50% | SMAD4 | 11, 07 | 253 | 28 | missense variant | NM 005359.5:c.608C>T | large intestine, thyroid |
| 16 | 60% | SMAD4 | 11, 59 | 138 | 16 | missense variant | NM 005359.5:c.1514T>C | large intestine |
| 21 | 90% | SMAD4 | 11, 65 | 309 | 36 | missense variant, splice region variant | NM 005359.5:c.953C>T | |
| 11 | 50% | SMARCB 1 | 13, 84 | 159 | 22 | missense variant, splice region variant | NM 003073.3:c.626A>C | |
| 11 | 50% | SMO | 15, 08 | 126 | 19 | missense variant | NM 005631.4:c.1025C>T | |
| 18 | 80% | SMO | 12, 7 | 126 | 16 | missense variant | NM 005631.4:c.1043C>T | |
| 11 | 50% | STK11 | 11, 29 | 124 | 14 | missense variant | NM 000455.4:c.793G>A | lung |
| 11 | 50% | STK11 | 11, 32 | 106 | 12 | missense variant | NM 000455.4:c.1048G>T | large intestine |

**ALT** The alternate allele
**Alt Variant Freq** The frequency of the Alt Allele
**Read Depth** The total number of reads passing quality filters at this position
**Alt Read Depth** The number of reads called at this position
**COSMIC Primary Site** The primary tissue type associated with the allele as reported in the COSMIC database.
[a] Human Genome Variation Society nomenclature
